# CD9-Positive Microvesicles Mediate the Transfer of Molecules to Bovine Spermatozoa during Epididymal Maturation

**DOI:** 10.1371/journal.pone.0065364

**Published:** 2013-06-13

**Authors:** Julieta N. Caballero, Gilles Frenette, Clémence Belleannée, Robert Sullivan

**Affiliations:** 1 Centre de Recherche du Centre Hospitalier Universitaire de Québec (CRCHU de Québec), Université Laval, Québec, PQ, Canada; 2 Centre de Recherche en Biologie de la Reproduction, Département d’Obstétrique, Gynécologie et Reproduction, Université Laval, Québec, PQ, Canada; Clermont-Ferrand Univ., France

## Abstract

Acquisition of fertilization ability by spermatozoa during epididymal transit occurs in part by the transfer of molecules from membranous vesicles called epididymosomes. Epididymosomes are heterogeneous in terms of both size and molecular composition. Exosomes and other related small membranous vesicles (30–120 nm) containing tetraspanin proteins on their surface are found in many biological fluids. In this study, we demonstrate that these vesicles are present in bovine cauda epididymal fluid as a subpopulation of epididymosomes. They contain tetraspanin CD9 in addition to other proteins involved in sperm maturation such as P25b, GliPr1L1, and MIF. In order to study the mechanism of protein transfer to sperm, DilC12-labeled unfractionated epididymosomes or CD9-positive microvesicles were coincubated with epididymal spermatozoa, and their transfer was evaluated by flow cytometry. CD9-positive microvesicles from epididymal fluid specifically transferred molecules to spermatozoa, whereas those prepared from blood were unable to do so. The CD9-positive microvesicles transferred molecules to the same sperm regions (acrosome and midpiece) as epididymosomes, with the same kinetics; however, the molecules were preferentially transferred to live sperm and, in contrast to epididymosomes, Zn^2+^ did not demonstrate potentiated transfer. Tetraspanin CD9 was associated with other proteins on the membrane surface of CD9-positive microvesicles according to coimmunoprecipitation experiments. CD26 cooperated with CD9 in the molecular transfer to sperm since the amount of molecules transferred was significantly reduced in the presence of specific antibodies. In conclusion, CD9-positive microvesicles are present in bovine cauda epididymal fluid and transfer molecules to live maturing sperm in a tissue-specific manner that involves CD9 and CD26.

## Introduction

After completing spermatogenesis and spermiation in the testis, mammalian spermatozoa are still unable to fertilize an oocyte; this ability is acquired during transit through the epididymis. Since sperm chromatin is condensed, transcription and translation are arrested, and sperm maturation depends on the interaction between the male gamete and the epididymal fluid microenvironment produced by secretion and reabsorption of inorganic and organic compounds by the epithelium [1], [2]. The most important maturational changes involve plasma membrane remodeling and the acquisition of forward motility. These changes are mainly the result of proteins and lipids acquired by the sperm plasma membrane [3], [4]. These macromolecules are synthesized by principal cells: some are secreted by merocrine pathways, whereas others are secreted by apocrine pathways. Apocrine secretion consists of the formation of apical blebs by the principal cells, and the release of membranous vesicles into the intraluminal fluid after breakdown of the apical blebs [5]. These membranous vesicles are referred to as epididymosomes [6]. They can be defined as membranous vesicles with a roughly spherical aspect and a bilayer membrane, and are heterogeneous in both size and content [7]. This heterogeneity could be explained by changes in the secretory characteristics of the principal cells along the epididymal duct [7]. It has been demonstrated that these membranous vesicles are able to transfer selected proteins involved in fertilization to the epididymal sperm under defined *in vitro* conditions [6]. However, little is known about the mechanisms involved in the interaction between epididymosomes and maturing spermatozoa. Zinc has been shown to potentiate protein transfer efficiency to the maturing spermatozoon [8].

In many biological systems, the intercellular communication mechanisms are mediated by the secretion and uptake of membranous vesicles. Cells are able to produce and secrete a wide variety of membranous vesicles into the extracellular space. These vesicles can be classified according to their size, functions, and cellular origin [9]. Exosomes and exosome-like vesicles have received the most attention in recent years. They are able to exchange proteins and lipids with different cell types, trigger downstream signaling events, and deliver specific nucleic acids [10]. They consist of small vesicles (30–120 nm) formed in endosomal compartments containing internal vesicles (multivesicular bodies) that store membrane-bound structures [9]. Sets of specific surface or adhesion molecules allow exosomes to target specific recipient cells [11], [12]. The protein family most commonly associated with exosomes is the tetraspanin family, specifically CD9, CD63, CD81, and CD82 [13]–[15]. It has been hypothesized that exosomes can fuse with the plasma membrane of recipient cells. CD9 is abundantly expressed in exosomes [16] and, even if non-fusogenic by itself, it could play a role in the formation of multimolecular complexes that will accomplish this function. Moreover, there is evidence that tetraspanins are organized into distinct microdomains called tetraspanin-enriched microdomains [17]. Tetraspanins can cluster other receptors, ligands, and fusogenic molecules through these domains, thereby regulating areas of cell–cell contact [18].

On the basis of these concepts, we propose that CD9-positive microvesicles are secreted into the intraluminal fluid as part of epididymosomes, a heterogeneous population of secreted membranous vesicles. We also hypothesize that their tetraspanin-enriched microdomains contain molecules that act as receptors for certain sperm ligands, which promotes contact and the transfer of molecules necessary for epididymal sperm maturation.

## Results

### Secretion of Exosomes in Distal Regions of the Epididymis

**Figure 1:**
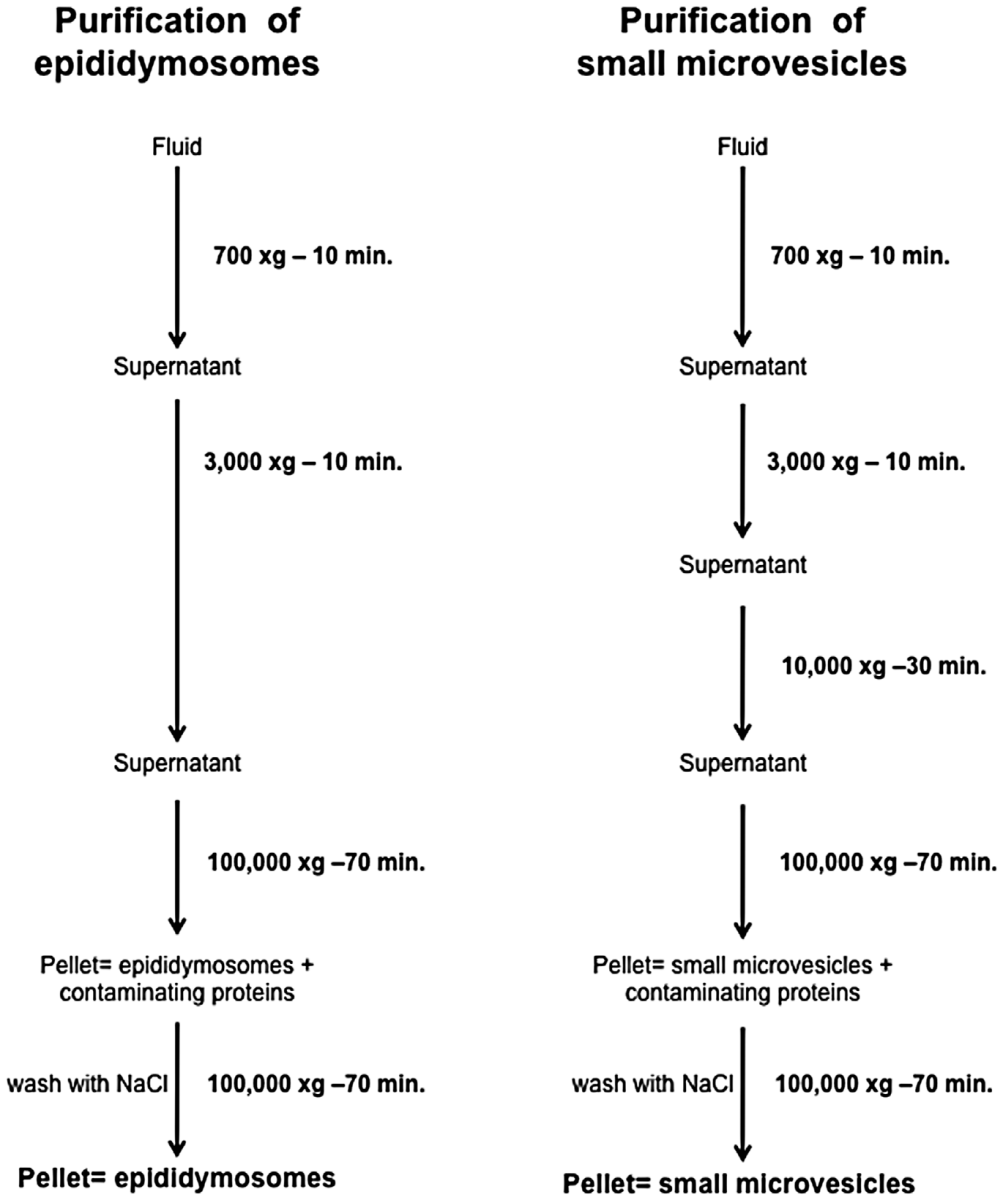
Protocols used to isolate epididymosomes or small microvesicles by differential centrifugation of epididymal fluids.

**Figure 2:**
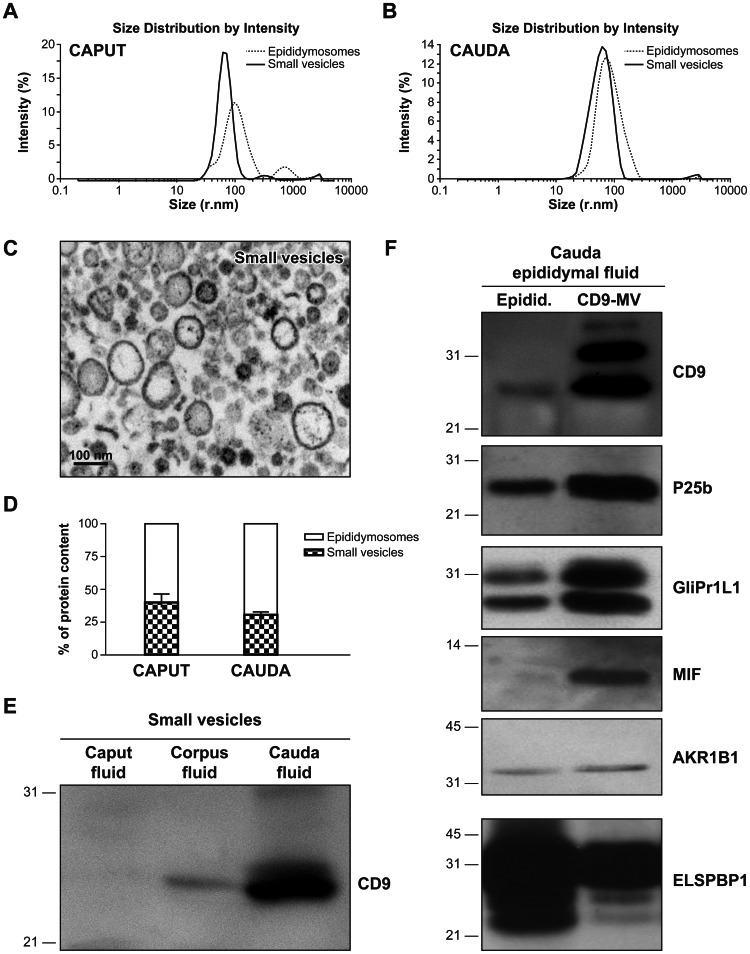
A population of small CD9-positive membranous vesicles with distinct protein content is present in the cauda epididymal fluid. Epididymosomes and small membranous vesicles were purified from caput (**A**) and cauda (**B**) epididymal fluid. Vesicle size was evaluated by Zetasizer NanoZS. The experiment was performed three times in duplicate. **C:** Cauda small membranous vesicle size, structure, and shape were evaluated by electron microscopy. Scale bar: 100 nm. **D:** Protein content of epididymosomes and small membranous vesicles purified from caput and cauda epididymal fluid was evaluated by the Bradford technique, and the proportion was calculated by considering epididymosomes as 100%. Measurements were performed in epididymal fluid from three different individuals. **E:** Western blot detection of CD9 (25 kDa) on Triton X-100 protein extracts from small membranous vesicles recovered from caput, corpus distal, and cauda epididymal fluid. Each lane contains the same amount of protein. Results are representative of three different experiments. **F:** Western blot detection of CD9 (25, 27, and 33 kDa), P25b, GliPr1L1, ELSPBP1, MIF, and AKR1B1 on Triton X-100 protein extracts from cauda epididymosomes (Epidid.) and CD9-positive microvesicles (CD9-MV). Each lane contains the same amount of protein. Molecular standards are indicated on the left side of the figure. Results are representative of three different experiments.

Membranous vesicles were purified from caput and cauda epididymal fluids by following a protocol that is routinely used to purify epididymosomes in our laboratory [8], or a protocol previously described to purify exosomes [19] (Fig. 1). Whereas the first protocol was designed to recover a heterogeneous population of microvesicles measuring between 20–1,000 nm and 25–300 nm in diameter in the caput (Fig. 2A) and cauda (Fig. 2B) respectively, the second protocol generates a more homogeneous population of vesicles with a diameter of 20–150 nm (Fig. 2A and B). This result was confirmed by electron microscopy (EM) (Fig. 2C). The small membranous vesicle population represents approximately 30% of the total proteins contained in the entire membranous vesicle population (epididymosomes) (Fig. 2D). CD9 is one of the most ubiquitous molecular markers for exosomes and its expression was evaluated on small membranous vesicle populations recovered from different epididymal regions by western blot. The protein was undetectable in caput, weakly expressed in the corpus, and highly expressed in the cauda region (25 kDa) (Fig. 2E). With reference to the fact that small vesicles (20–150 nm) purified from the cauda epididymal fluid express CD9, this small membranous vesicle population recovered from the cauda epididymal fluid will henceforth be referred to as CD9-positive microvesicles. In support of this statement, it was found that the level of expression of CD9 (detected as a triple band at 25, 27, and 33 kDa by western blot) was higher in this population than in unfractionated epididymosomes also recovered from the cauda epididymal fluid (Fig. 2F). The presence of proteins known to be transferred from membranous vesicles to spermatozoa during epididymal transit was also observed and analyzed. P25b, GliPr1L1, and MIF were highly expressed in CD9-positive microvesicles, AKR1B1 was detected at similar levels in both microvesicle populations, and ELSPBP1 was more abundant in the epididymosomal population (Fig. 2F). On the basis of these results, we can conclude that CD9-positive microvesicles are predominantly found in the epididymal fluid that is recovered from the cauda region.

**Figure 3:**
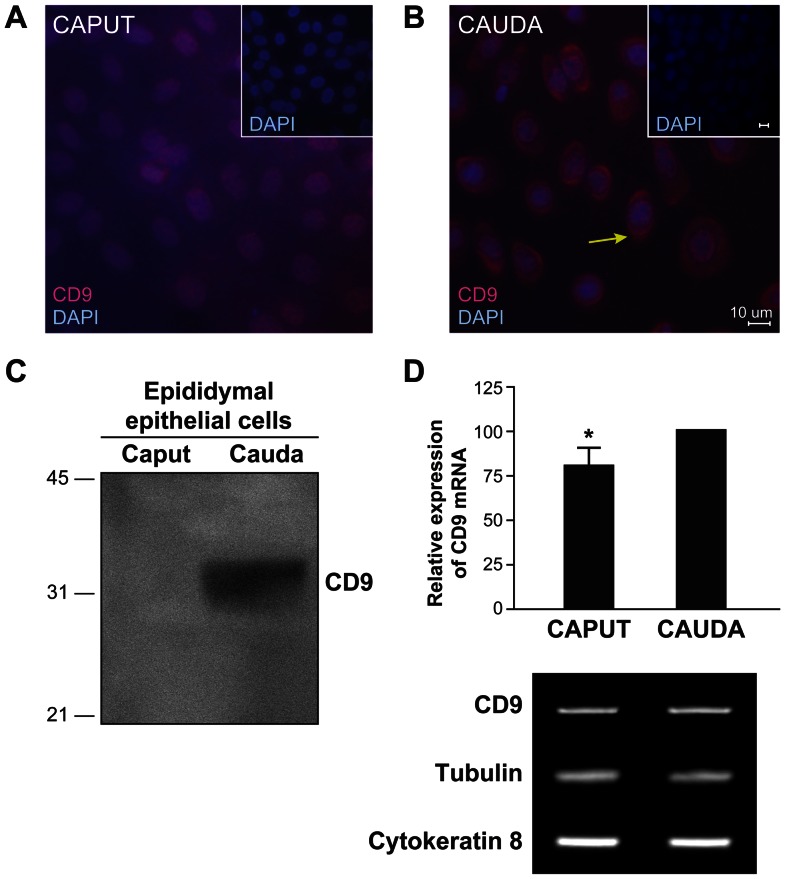
The exosome marker CD9 is highly expressed by cauda epididymal epithelial cells in intracellular structures. Immunolocalization of CD9 (red) in caput (**A**) and cauda (**B**) epididymal epithelial primary cells cultured *in vitro*. Nuclei were counterstained with DAPI (blue). Yellow arrow indicates intracytoplasmic localization. Scale bar: 10 µm. **C**: Western blot detection of CD9 (25 kDa) on Triton X-100 protein extracts of epididymal tubules dissected from the caput and cauda epididymal regions. Each lane contains the same amount of protein. Results are representative of at least three different experiments. **D**: Semi-quantitative analysis of the expression of CD9 using tubulin as a housekeeping gene. The highest expression was considered to be 100%, results are presented as average ± s.e.m. from three different experiments, * differs significantly *p*<0.05. Dissected and digested epididymal caput and cauda tubules and epithelial cells showed comparable amounts of total (tubulin) and epithelial cell cytokeratin 8 transcripts.

**Figure 4:**
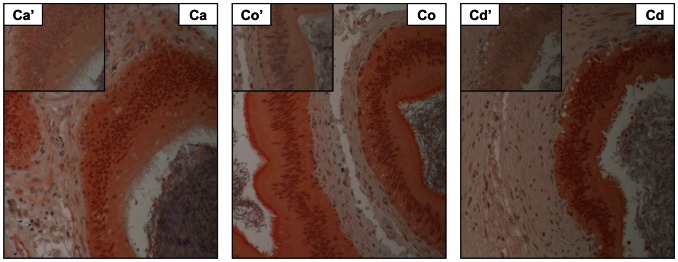
Localization of CD9 in the epididymal duct. Immunohistological localization of CD9 in different sections of the bovine epididymis: caput (Ca), corpus (Co), and cauda (Cd). Inserts represent negative controls. Immune complexes are observed in red and nuclei are counterstained in blue.

Since exosomes secreted by different cell types originate from intracellular multivesicular bodies, the expression of CD9 was studied in epididymal epithelial cells. CD9 was weakly expressed in the perinuclear region of primary epithelial cell cultures recovered from both the caput and cauda regions (Fig. 3A, 3B); however, CD9 was also immunolocalized in the cytoplasm of epithelial cells from the cauda (Fig. 3B). The presence of CD9 (32 kDa) was also detected in protein extracts from cauda epididymal epithelial cells (Fig. 3C), but not from cells recovered from the caput. The transcript encoding CD9 was found in epididymal epithelial cells from both the caput and cauda regions, with higher expression in the cauda (Fig. 3D). In agreement with these results, the expression of CD9 was observed in epithelial cells of caput, corpus and cauda epididymal regions, with a higher level of expression in the apical borders of the cauda region (Fig. 4).

### Transfer of Molecules from Membranous Vesicles to Epididymal Spermatozoa

**Figure 5:**
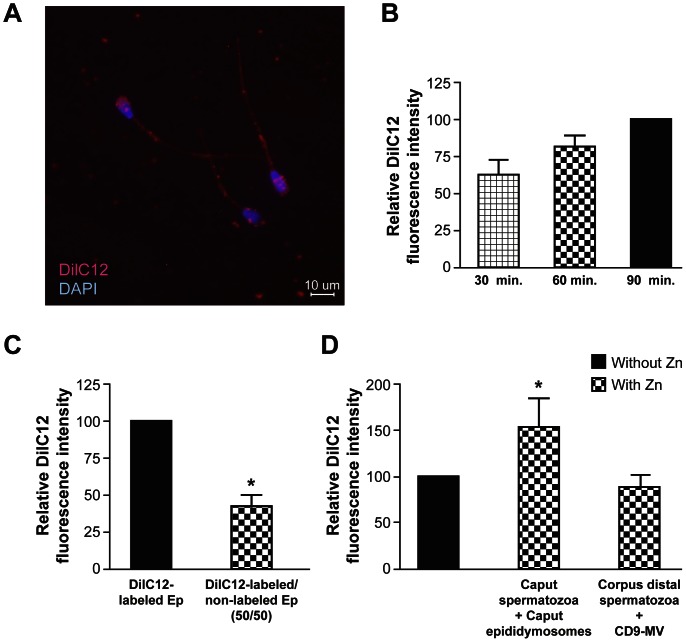
Membranous vesicle–sperm molecular transfer mechanism. **A:** CD9-positive microvesicles DilC12-labeled molecule localization on corpus distal epididymal sperm after coincubation for 1 hour at 37°C in sperm medium at pH 6.5. Similar results were obtained with sperm incubated with labeled epididymosomes (data not shown). **B:** Relative DilC12 fluorescence intensity on live corpus distal epididymal sperm after coincubation with cauda epididymosomes for 30, 60, and 90 min. **C:** Relative DilC12 fluorescence intensity on live corpus distal epididymal sperm after coincubation with cauda epididymosomes labeled with DilC12 (DilC12-labled Ep) or DilC12-labeled cauda epididymosomes diluted 1∶1 with non-labeled epididymosomes (DilC12-labeled/non-labeled Ep) after 60 min of coincubation. **D:** Relative DilC12 fluorescence intensity on live caput epididymal sperm after coincubation with cauda epididymosomes and on corpus distal epididymal sperm after coincubation with CD9-positive microvesicles (CD9-MV) labeled with DilC12 for 60 min in the presence or absence of 1 mM ZnCl_2_ (Zn). In all cases, the maximum fluorescence was considered to be 100%. Results are presented as average ± s.e.m., * differs significantly *p*<0.05. Experiments were performed at least three times.

Epididymosomes or CD9-positive microvesicles were labeled with the membrane lipid probe DilC12 and coincubated with epididymal spermatozoa. Labeled membrane molecules were transferred from both epididymosomes and CD9-positive microvesicle preparations to specific regions of the spermatozoa *i.e.*, acrosome and midpiece (Fig. 5A). The intensity of labeling increased with time of coincubation up to 90 min (Fig. 5B). Longer incubation times affected viability. When DilC12-labeled epididymosomes or CD9-positive microvesicles were diluted with an unlabeled population of the same vesicles during coincubation experiments, the transfer of DilC12 decreased to 42.2±8.9% or 51.7±1.0%, respectively (Fig. 5C) indicating a competition effect.

Human exosomes are well-characterized examples of CD9-positive microvesicles [20]. If specific molecules are involved in the recognition, adhesion, and/or fusion processes in bovine epididymal microvesicles expressing CD9, it is expected that only these vesicles will transfer molecules to the sperm, and not those of human origin. When CD9-positive (Fig. S1A) human blood serum exosomes were evaluated by EM, we observed that they exhibited similar structural characteristics to bovine epididymal small microvesicles (data not shown). Hence, these vesicles were tested for their ability to transfer molecules to sperm. When coincubated with epididymal spermatozoa, the human exosomes transferred a significantly lower quantity of molecules compared with CD9-positive microvesicles recovered from bovine epididymal fluid (Fig. S1B). Furthermore, they did not exhibit the same specific interaction with sperm subcellular domains (Fig. S1C) as vesicles of epididymal origin (Fig. 5A).

Since Zn^2+^ enhances protein transfer from cauda epididymosomes to the caput sperm in coincubation experiments [8], the effect of this cation was evaluated in coincubation experiments between caput or distal corpus epididymal spermatozoa with epididymosomes or CD9-positive microvesicles. The presence of Zn^2+^ increased sperm DilC12 labeling when caput spermatozoa were incubated with caput epididymosomes (Fig. 5D). Similar results were observed when the incubation was performed in the presence of cauda epididymosomes or CD9-positive microvesicles (data not shown). However, when cauda epididymal sperm were incubated with cauda epididymosomes (data not shown) or CD9-positive microvesicles (Fig. 5D), the addition of Zn^2+^ had no significant effect. These results indicate that the Zn^2+^-dependent transfer mechanism is associated with caput sperm and does not participate in the mechanism of transfer between CD9-positive microvesicles and cauda epididymal sperm.

**Figure 6:**
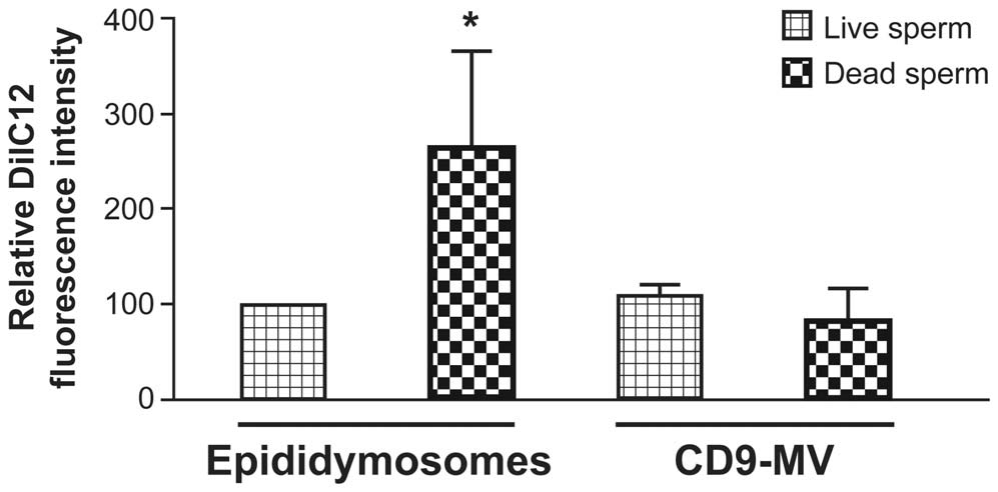
Epididymosomes have more affinity to transfer molecules to dead sperm. Relative DilC12 fluorescence intensity on live and dead corpus distal epididymal sperm after 60 min of coincubation with cauda epididymosomes or CD9-positive microvesicles (CD9-MV) labeled with DilC12. The maximum fluorescence of the control was considered to be 100%. Results are presented as average ± s.e.m. from three different experiments, * differs significantly *p*<0.05.

Despite similarities in the kinetic incorporation and localization of the transferred molecules when sperm were coincubated with epididymosomes or CD9-positive microvesicles, epididymosomes showed a significantly higher ability to transfer molecules to dead sperm when compared to CD9-positive microvesicle preparations (Fig. 6).

### Tetraspanin-enriched Microdomains are Involved in the Mechanism of Molecular Transfer

Cell adhesion and membrane fusion have been linked to tetraspanin molecules and their membrane domains [21]. CD9 and associated molecules in epididymal CD9-positive microvesicles were thus evaluated for their ability to promote the transfer of molecules to spermatozoa. In order to determine the molecules associated with CD9 in the tetraspanin-enriched microdomains, CD9-positive microvesicles were extracted with Brij O10 to conserve protein–protein interactions in the tetraspanin webs, followed by immunoprecipitation with anti-CD9 antibodies. Six proteins that specifically immunoprecipitated with CD9 were identified by LC/MS–MS: gamma-glutamyltransferase 1-like/CD224, dipeptidyl peptidase 4/CD26, prominin 2-like/CD133, clusterin, N-acetylated-alpha-linked acid dipeptidase 2 (predicted protein), and ectonucleotide pyrophosphatase/phosphodiesterase family member 3 (Table 1).

**Table 1:** LC–MS/MS identification of proteins that coimmunoprecipitated with CD9 after BrijO10 protein extraction of CD9-positive microvesicles.

Proteins specifically associated with CD9				
	Protein	Accession number	Molecular Weight (kDa)	Number of peptides (min peptide 95%)
1	PREDICTED: gamma-glutamyltransferase 1-like/CD224	UPI0000EBD9A3	61	21
2	DPP4_BOVIN Dipeptidyl peptidase 4/CD26	P81425	88	4
3	PREDICTED: prominin 2-like/CD133	UPI0001D57626	99	4
4	CLUS_BOVIN Clusterin	P17697	51	3
5	F1MGS8_BOVIN Uncharacterized protein (Fragment)	F1MGS8	83	2
6	ENPP3_BOVIN Ectonucleotide pyrophosphatase/phosphodiesterase family member 3	P15396	100	2

**Figure 7:**
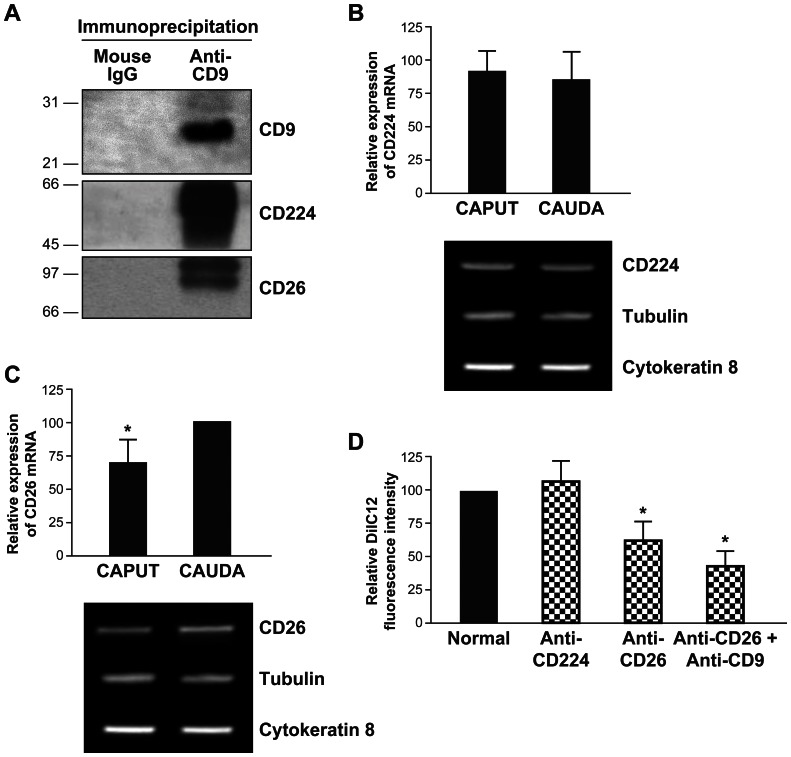
CD26 is associated with CD9 in microvesicles and has a synergistic role with CD9 in the molecular transfer to the corpus distal sperm. **A:** CD9-positive microvesicles were lysed in presence of BrijO10, CD9 (25 kDa) was immunoprecipitated and analyzed by western blot. CD26 (110 kDa) and CD224 (61 kDa) coimmunoprecipitated with CD9. Normal mouse IgG was used as the negative control. Semi-quantitative analysis of RNA expression of CD224 (**B**) and CD26 (**C**) in epididymal epithelial cells, using tubulin as the housekeeping gene and cytokeratin 8 as a marker of epithelial cells. The highest expression was considered to be 100%, results are presented as average ± s.e.m. from three different experiments, * differs significantly *p*<0.05. Primary cultures of epithelial cells from caput and cauda epididymal tubules show comparable amounts of total (tubulin) and epithelial cell (cytokeratin 8) transcripts. **D:** Relative fluorescence intensity on live corpus distal epididymal sperm after 60 min coincubation with DilC12-labeled CD9-positive microvesicles in the presence of normal IgG or anti-CD224, anti-CD26 or anti-CD9 and anti-CD26 antibody Fab fragments. The maximum fluorescence was considered to be 100%. Results are presented as average ± s.e.m. from three different experiments, * differs significantly *p*<0.05.

Since CD26 and CD224 were previously found to be associated with tetraspanin webs in cell plasma membranes [22], [23], their involvement in sperm interaction was studied further. Their association with CD9 on CD9-positive microvesicles was corroborated by immunoprecipitation and western blot (Fig. 7A). Analysis of their expression in epididymal epithelial cells from caput and cauda revealed that CD224 was homogenously expressed at the mRNA level in both proximal and distal regions (Fig. 7B), whereas CD26 expression was significantly higher in the cauda epididymis (Fig. 7C) and followed the same pattern as CD9 mRNA (Fig. 3D).

When corpus distal spermatozoa were incubated with DilC12-labeled cauda epididymosomes in the presence of anti-CD9 antibodies, high concentrations of Fab fragment were necessary to inhibit transfer by 10–15% when compared to control Fabs (data not shown). This effect was not observed when caput spermatozoa were incubated with caput epididymosomes (data not shown), which is in agreement with the absence of CD9 in membranous vesicles from the caput epididymal fluid (Fig. 2E). When corpus distal spermatozoa were incubated with CD9-positive microvesicles in the presence of anti-CD224 or anti-CD26 Fab antibody fragments, a significant decrease in sperm DilC12 labeling was found only in the presence of the anti-CD26 antibodies (42.5±12.0%, Fig. 7D). This inhibitory effect was enhanced when anti-CD9 and anti-CD26 Fabs were used in combination (61.5±15.0%); thus, suggesting a cooperative effect between the two molecules (Fig. 7D).

## Discussion

Exosomes are small membranous vesicles that are secreted by a wide range of mammalian cell types, and transfer different molecules to a distant target cell. Principal cells in the epididymal epithelium produce membranous vesicles as a product of their apocrine secretion. However, it has been reported that this mode of secretion varies along the epididymal duct in the mouse [7]. Indeed, in proximal epididymal segments, the presence of multivesicular bodies containing small membranous vesicles is observed, which resembles exosome secretion [7]. The presence of multivesicular bodies was previously reported in the bovine epididymis [24]. Despite examination at the EM level, there is no evidence in the literature that supports the concept of fusion of these ultrastructures with the apical membrane of epididymal principal cells. Furthermore, there is no evidence that CD9-positive microvesicles found in the epididymal intraluminal compartment originate from these structures. Although the microvesicle secretion pattern along the bovine duct has not been studied in great detail, we recently reported that epididymosomes recovered from the caput and cauda regions are heterogeneous in size and content [25]. In the present study, we show that bovine caput epididymosomes exhibit more variation in size than cauda epididymosomes, suggesting that a secretion pattern variation exists along the excurrent duct. Size is not the only criterion used to define the origin of a microvesicle found in biological fluids; composition also has to be taken into account. Tetraspanin CD9 and CD63 are the most common exosome markers [26]. When applied to fluids collected from the caput and cauda epididymal regions, the protocol used to isolate exosomes generates a homogeneous population of small membranous vesicles [19]. Only small microvesicles secreted from the corpus distal to the cauda regions were CD9-positive. This is in agreement with our previous proteomic analysis of epididymosomes identifying CD9 associated with cauda epididymosomes [25]. This CD9-positive microvesicle secretion in the distal regions of the epididymis is supported by immunodetection of a 33 kDa protein band using an anti-CD9 antibody, as well as significantly higher expression of CD9 transcripts in cauda compared with caput epididymal epithelial cells, and the presence of protein in the cytoplasmic compartment of the cauda epididymal epithelial cells. Differences in molecular weights of CD9 in protein extracts from epididymal epithelial cells are probably a result of posttranslational modifications such as glycosylation and palmitoylation during tetraspanin-enriched microdomain formation prior to CD9-positive microvesicle secretion [27], [28].

CD9-positive microvesicles represent about one third of the total proteins contained in the vesiculated fraction of the epididymal fluid. Among the more abundant proteins detected in this vesicle population are P25b, GliPr1L1, and MIF. Of particular interest is that all three proteins are important for sperm physiology [29]–[32]. On the other hand, we have recently demonstrated that ELSPBP1, which is more abundant in larger sized microvesicles, is added to dead sperm and not to sperm that die during incubation [33]. This result is in agreement with the finding that epididymosomes show more affinity for the transfer of molecules to dead rather than live sperm, whereas CD9-positive microvesicles exhibit the opposite behavior. These results clearly demonstrate the existence of a heterogeneous population of vesicles with different composition, origin, and function in epididymal fluid.

Use of the DilC12 fluorescent probe revealed that molecules from epididymosomes and CD9-positive microvesicles are incorporated into the plasma membrane of spermatozoa after *in vitro* coincubation at 37°C/pH 6.5 under conditions that mimic the intraluminal compartment of the bovine epididymis. The fact that labeled vesicles transfer less molecules to sperm in the presence of a population of non-labeled vesicles indicates the existence of a competition effect. This effect suggests that the incorporation is saturable, and the presence of specific regions and/or receptors for this mechanism to occur. The incorporation of molecules occurs mainly within the first 30 min of incubation, and some sperm subcellular regions incorporate the microvesicular molecules with more affinity than others; for example, the acrosome and midpiece. Epididymosomes and CD9-positive microvesicles transfer molecules to the same regions of spermatozoa; however, CD9-positive microvesicles are enriched with a different subset of proteins when compared with epididymosomes. Accordingly, we propose that CD9-positive microvesicles transfer GliPr1L1 and P25b to the acrosomal region, which is involved in sperm–egg interactions, whereas epididymosomes transfer ELSPBP1 to this region [33]. In this work, we demonstrate that the presence of Zn^2+^ affects the molecular transfer of microvesicles to the caput spermatozoa, but not to the cauda epididymal sperm. This observation, coupled with the specific localization of the transferred molecules, allows us to propose that the transfer is coordinated by ligands present on the sperm surface, which require specific receptors on the microvesicle membranes.

Tetraspanin-enriched microdomains have been proposed to play a role in the adhesion of exosomes to their target cells [34]–[36]. In the present work, we demonstrated that the transfer of molecules from CD9-positive microvesicles to corpus distal sperm decreases when CD9 is blocked by specific antibodies. However, this blockage was not biologically relevant (10–15%). This finding may be explained by the fact that this molecule is not fusogenic by itself; rather, it is organizes complexes of other fusogenic molecules. CD9 is a molecular component of tetraspanin webs. In these domains, tetraspanins are primarily and secondarily associated with other transmembrane proteins [37]. Other molecules such as CD26 [22] and CD224 [23], also known to be associated with different cell types, were found to be associated with CD9 in tetraspanin webs of CD9-positive microvesicles. In order to investigate the role of these molecules in the transfer of CD9-positive microvesicles to spermatozoa during epididymal maturation, the same experimental approach was carried out. The results revealed that CD26, but not CD224, had a synergic effect with CD9. Concomitant with these findings, CD26, but not CD224, was found to be expressed in a similar manner to CD9 along the epididymal epithelium. In support of these results, CD224 has been found to be expressed in the apical plasma membrane of the epithelium along the epididymis as part of the cell antioxidant defense mechanism [38]; thus suggesting that this microvesicular molecule does not have a functional role in adhesion. In contrast, CD26 has been shown to enhance the molecular transfer from prostasomes to epididymal spermatozoa in the stallion [39], [40], and is associated with CD9 in the tetraspanin web of cancer cell lines when they became invasive [22]. Other proteins found in the exosome tetraspanin-enriched microdomains were CD133, ectonucleotide pyrophosphatase/phosphodiesterase family member 3, and clusterin. CD133 has been found to be associated with microvilli as well as small membrane particles that are released into different physiological fluids [41]. Ectonucleotide pyrophosphatase/phosphodiesterase family member 3 has been described in the proteomic analysis of other exosomes [42], [43]. The presence of clusterin has been observed in epididymal fluid: potential roles in tissue remodeling and apoptosis have been proposed, in addition to sperm cholesterol homeostasis, which facilitates plasma membrane remodeling during epididymal maturation [2].

When membranous vesicles probed with DilC12 were coincubated with epididymal spermatozoa, fluorescence was transferred to sperm subcellular domains, suggesting that lipids were transferred from the membranous vesicles to the sperm during coincubation. Moreover, changes in lipid composition and fluidity occur simultaneously in membranous vesicles and spermatozoa in a given epididymal region, suggesting that there is a transfer of lipids during the epididymal transit of sperm [7]. On the basis of these findings, we propose that tetraspanin-enriched microdomains in the epididymal CD9-positive microvesicles play a role in lipid transfer in addition to protein transfer during epididymal sperm maturation.

In conclusion, sperm maturation and plasma membrane remodeling during epididymal transit depend on the epididymal fluid microenvironment. Apocrine secretion plays an important role in this function with the delivery of membranous vesicles in the intraluminal fluid. In the present work, we demonstrate that different kinds of microvesicles are produced along the epididymal duct. The secretion of CD9-positive microvesicles occurs in the distal sections of the tubule. These vesicles contain tetraspanin-enriched microdomains in their membranes. These domains contain molecules with important adhesion and/or fusion functions. These molecules form a multimolecular complex that favors the transfer of lipids and proteins from the microvesicle membrane to the sperm membrane. In addition to this function, tetraspanin-enriched microdomains contain other molecules with potential roles in oxidative stress protection of the sperm. In summary, we propose that CD9-positive microvesicle production in the distal region of the epididymis plays an important role in the acquisition of sperm fertilization ability, and tetraspanin-enriched microdomains are one of the molecular complexes that drive the cell–cell communication between epididymal microvesicles and spermatozoa.

## Materials and Methods

### Recovery of Epididymal Fluids and Human Blood Serum

Testes and epididymides from mature bulls were obtained from the Colbex abattoir (St-Cyrille de Wendover, QC, Canada) and were transported to the laboratory on ice. Upon arrival, epididymides were dissected and epididymal fluids from caput, corpus and cauda sections were obtained as previously described by Frenette *et al.* [8]. In brief, caput and corpus distal fluids were obtained by cutting the tubules and applying pressure to their proximal portion. Cauda fluid was collected by retrograde flushing by applying air pressure into the vas deferens. After resuspension in PBS (137 mM NaCl, 3 mM KCl, 8 mM Na_2_HPO_4_, and 1.5 mM KH_2_PO_4_, pH 7.3), epididymal fluids were centrifuged at 700×g for 10 min. Pelleted spermatozoa were washed with PBS. Supernatants were centrifuged at 3,000×g for 10 min to remove cell debris, and then processed for epididymosomes or small vesicle purification.

Venous blood from healthy volunteers was collected on isocitrate anticoagulant solution and centrifuged at 250×g for 10 min. The resulting platelet-rich plasma was subjected to a second centrifugation at 3,000×g for 10 min to remove cell debris, and then processed for small vesicle purification.

### Membranous Vesicle Purification, Size and Structural Evaluation

The supernatant from epididymal fluid was subjected to epididymosome purification by two ultracentrifugations at 100,000×g for 70 min. Alternatively, small vesicles were purified by performing an additional centrifugation (10,000×g for 30 min) prior to the two ultracentrifugations [19]. This procedure was also used to isolate human blood serum small membranous vesicles (Fig. S1). Protein concentration was determined using the Bio-Rad protein assay kit (Bio-Rad Laboratories, Hercules, CA). Particle size was determined by evaluating the Brownian motion of microvesicles using a Zetasizer Nano Series analyzer (ZEN 3600, Malvern Instruments, Malvern, Worcestershire, UK). Samples were diluted in PBS and measured at 4°C. Data were analyzed using Zetasizer software 6.20. Samples were measured three times, and the average and standard deviation were determined. Structural and purity analysis of different membranous vesicles preparations were evaluated by EM. Pellets containing membranous vesicles were fixed for 1 h at 4°C in 4% paraformaldehyde, 0.25% glutaraldehyde supplemented with 100 mM Na-cacodylate and 2 mM CaCl_2_. The fixative solution was removed by dilution in 150 mM NaCl and ultracentrifugation. The specimens were placed on Ni/Formvar grids and colored with PTA (phosphotungstic acid). Analyses were performed on a JEOL, JEM -1230 transmission electron microscope at 80 kV (JEOL, Montreal, QC, Canada).

### Epididymal Sperm-membranous Vesicle Coincubations

For fluorescence labeling, membranous vesicles were incubated for 15 min at 37°C with the DilC12 (D-383, Invitrogen, Carlsbad, CA, USA) membrane probe (equivalent of 120 µg proteins of membranous vesicles in 50 µl with 25 µl of a 20 µM DilC12 solution in 2% ethanol). Unbound probe was removed by washing the microvesicles with 150 mM NaCl by ultracentrifugation. The resulting pellet was resuspended in sperm medium (10 mM MES buffer, pH 6.5 supplemented with 114 mM NaCl, 3.1 mM KCl, 25 mM NaHCO_3_, 0.3 mM NaH_2_PO_4_, 10 mM sodium lactate, 2 mM CaCl_2_, 0.5 mM MgCl_2_, 0.2 mM sodium pyruvate, 10 mM D-glucose, and 1 mg/mL polyvinyl alcohol).

Thirty million washed spermatozoa from caput or corpus distal epididymal regions were resuspended in 50 µl of sperm medium and incubated with 30 µg of DilC12-labeled membranous vesicles for 60 min at 37°C under different experimental conditions *i.e.*, in the presence or absence of 1 mM ZnCl_2_, or in the presence of 7.5 or 15 µg/ml of different IgG Fab fragments: normal IgG, anti-CD9 (MA1-19301, Thermo Scientific, Rockford, IL, USA), anti-CD26 (YV0244-01, Accurate Chemical and Scientific Corporation, Westbury, NY, USA), or anti-CD224 (ST1551, EMD Millipore, Billerica, MA, USA). Fab fragments were digested and purified using Pierce Fab Micro Preparation kit (44685, Thermo Scientific) After washing, spermatozoa were incubated for a further 10 min with LIVE/DEAD Fixable Near-IR Dead Cell Stain (L10119, Invitrogen) according to D’Amours *et al.* [44]. Spermatozoa were fixed with 3.7% formaldehyde for 15 min and incubated with 5 mg/ml Hoechst 33342 (H-3570, Invitrogen) after washing. Sperm suspensions were analyzed by flow cytometry (BD FACSAria-II; BD Biosciences, Mississauga, ON). Fluorescent probes were detected using 375 nm laser and 450/50 band pass filter (Hoechst 33342), 532 nm laser and 585/12 band pass filter (DilC12), and 633 nm laser and 780/60 band pass filter (LIVE/DEAD Fixable Near-IR Dead Cell Stain).

### Isolation of Epididymal Epithelial Cells and Cell Culture

Caput and cauda epididymal epithelial cells were prepared as previously described [45]. In brief, each epididymal section was dissected, freed from connective tissue, and cut into small pieces of 1–2 mm^3^. In order to remove epididymal fluid and spermatozoa, the tissue samples were then rinsed several times with a saline solution (150 mM NaCl containing 10 IU/ml penicillin and 100 mg/ml streptomycin) and digested in serum-free Epididymal Cell Medium (ECM [46]), containing 2.5 mg/ml collagenase type II in a shaking bath at 37°C for 1 h. Collagenase-dispersed cell clusters were cultured in a 6-well dish with cover slips on the well bottom in ECM supplemented with 10% FBS in a humidified atmosphere containing 5% CO_2_ at 37°C. Confluence was reached after 5 to 7 days of culture and more than 90% of cultured cells were of epithelial origin (evaluated by probing epithelial cadherin plasma membrane with specific antibodies).

### Immunolocalization of CD9 on Spermatozoa, Epididymal Epithelial Cells and Epididymal Tissues

Washed epididymal spermatozoa from caput and cauda epididymal sections were smeared onto slides and fixed with 3.7% formaldehyde in PBS. Coverslips containing confluent epididymal epithelial cells were fixed with methanol at −20°C for 10 min and permeabilized in 0.3% (v/v) Triton X-100 in PBS for 15 min. Nonspecific sites were blocked with 1% BSA in PBS for 1 hour. Slides were then incubated overnight at 4°C with 10 µg/ml of polyclonal anti-CD9 antibodies (H-110, Santa Cruz Biotechnologies Inc., Santa Cruz, CA, USA) or normal rabbit IgG as control in 0.5% of goat serum in PBS. Unbound antibodies were washed with PBS, and the secondary antibodies, anti-rabbit IgG Alexa 488 (green) or 568 (red)-conjugated antibodies, were incubated for 1 h at room temperature in the dark. Samples were mounted with Vectashield supplemented with DAPI (Vector Laboratories, Burlingame, CA, USA) and observed under a Zeiss Axioskop 2 epifluorescence microscope (Carl Zeiss Canada, Toronto, ON, Canada).

Epididymal tissues were fixed in PBS containing 4% paraformaldehyde for 3 days at 4°C and then embedded in paraffin. Thin sections of 6 microns were rehydrated, treated for 20 min in 3% H_2_O_2_ in methanol, washed in PBS, and boiled for 10 min in a 10 mM citrate solution pH 6.0. The sections were blocked with 6.5% goat serum in PBS for 2 h, then incubated with 10 µg/ml of polyclonal anti-CD9 antibodies (H-110, Santa Cruz Biotechnologies Inc., Santa Cruz, CA, USA) or normal rabbit IgG as control for sixteen hours at 4°C, followed by incubation with biotinylated goat anti-rabbit IgG. Detection was performed with ABC Vectastain kit (Vector Laboratories, Burlingame, California). Nuclei were counterstained with Hematoxylin.

### PCR

Total RNA was extracted by homogenizing epididymal epithelial cells with TRIzol reagent after collagenase dispersion (Invitrogen Life Technologies, CA, USA), precipitated with CHCl_3_/isopropanol, and purified with the RNeasy Mini Kit (Qiagen, Chatsworth, CA) according to manufacturers’ instructions. After reverse transcription, tubulin, cytokeratin 8, CD9, CD26, and CD224 were amplified by polymerase chain reaction (PCR). Primers and product length for each gene are described in Table S1. Expression data was normalized to tubulin as a housekeeping gene.

### Protein Extraction and Western Immunoblotting

Caput and cauda epididymal sperm or their cavitated membranes, membranous vesicles, epididymal epithelial cells after collagenase dispersion, or spermatozoa after incubation with membranous vesicles were subjected to 1% (v/v) Triton X-100 protein extraction in 10 mM Tris buffer (pH 7.4) containing 150 mM NaCl, 1 mM CaCl_2_, and 1 mM MgCl_2_. After extraction, proteins were precipitated with acetone at −20°C for 2 h and solubilized in Laemmli sample buffer, without ß-mercaptoethanol in case of CD9 detection. Proteins were subjected to 10 or 12% SDS-PAGE and electrotransferred onto nitrocellulose membrane. Membranes were blocked with 5% dried skimmed milk in PBS supplemented with 0.1% (v/v) Tween-20 for 1 h and incubated with anti-CD9 (LS-C45127, LifeSpan Biosciences Inc., Seattle, WA, USA), anti CD26, or anti CD224 antibodies for 2 h. Additional antibodies used included: P25b protein (antisera produced in our laboratory [29]), macrophage migration inhibitory factor (MIF, from Dr M. Nishibori), aldose reductase AKR1B1, a gift from Dr. M.A. Fortier [47], anti-recGliPr1L1 [30], or anti ELSPBP1 antisera [33]. After washing, membranes were incubated for 1 h with goat anti-mouse or anti-rabbit IgG coupled to horseradish peroxidase. In the case of biotinylated proteins, the membrane was blocked and then incubated with NA-HRP (neutravidin-conjugated horseradish peroxidase; Pierce) for 90 min in 1% dried skimmed milk in PBS-Tween. Horseradish peroxidase complexes were revealed using a chemiluminescent peroxidase substrate (Amersham, Buckinghamshire, UK).

### Coimmunoprecipitation and LC/MS-MS Analysis

CD9-associated molecules were identified by lysing 3–4 mg of cauda epididymal small membranous vesicles in 1 ml of lysis buffer 1% Brij O10 (P6136, Sigma, St. Louis, MO, USA) in 10 mM Tris pH 7.4, 150 mM NaCl, 1 mM CaCl_2_, and 1 mM MgCl_2_ for 30 min at 4°C. Insoluble material was removed by centrifugation at 12,000×g for 15 min at 4°C and the supernatant was precleared for 1 h at 4°C with 20 µl protein G-sepharose beads (17-0618-01, GE Healthcare, Upsala, Sweden) previously blocked overnight at 4°C with 50 mM Tris buffer (pH 8.0) supplemented with 200 mM ethanolamine and 1% (v/v) Tween-20. Total lysate was divided into two equal volume aliquots; 2 µg of anti-CD9 antibodies (LS-C45127, LifeSpan Biosciencies Inc.) were added to one aliquot while normal mouse IgGs were added to the control lysate and incubated overnight at 4°C. Immunocomplexes were precipitated with 10 µl of protein G-sepharose beads for 1 h at 4°C. After washing the beads with lysis buffer, the coprecipitated molecules were eluted with lysis buffer supplemented with 1% (v/v) Triton X-100 and 0.2% (w/v) SDS. Extracted proteins were precipitated with acetone at −20°C, and resuspended in Laemli sample buffer without ß-mercaptoethanol. Immunoprecipitates were separated by 5–15% gradient SDS-PAGE under non-reducing conditions, and stained with Coomassie blue. Specific bands immunoprecipitated with anti-CD9 antibodies were excised, digested with trypsin, and analyzed by the Proteomics Platform of the Eastern Quebec Genomics Center, Quebec, Canada. Peptide samples were separated by online reversed-phase (RP) nanoscale capillary liquid chromatography (nanoLC) and analyzed by ES–MS/MS. All MS/MS samples were analyzed using Mascot (Matrix Science, London, UK; version 2.2.0). Mascot was set up to search the bovine Uniref100 database (release 11.05) assuming digestion with trypsin. Mascot was searched with a fragment ion mass tolerance of 0.50 Da and a parent ion tolerance of 2.0 Da. Scaffold (version Scaffold 3.0.9.1, Proteome Software Inc., Portland, OR, USA) was used to validate MS/MS-based peptide and protein identifications. The criterion to identify a protein was the content of at least two unique peptides identified with 95% probability.

### Semi-quantitative and Statistical Analysis

For semi-quantitative analysis of protein or mRNA expression, films or agarose gels were scanned and images were analyzed and quantified using Adobe Photoshop CS2 version 9.0.2 software using the previously described method (http://www.lukemiller.org/journal/2007/08/quantifying-western-blots-without.html). Semi-quantitative determination of mRNA expression was performed using tubulin as a loading control. For semi-quantitative analysis of relative DilC12 fluorescence intensity, control conditions (normal mouse IgG) were considered to be 100%. At least three replicates were performed for each set of experiments. Results were expressed as average ± s.e.m. Data were analyzed by paired Student’s *t*-tests where controls were compared with each treatment condition. A difference with *p*<0.05 was considered significant.

## Supporting Information

Figure S1 **Human blood serum exosomes fail to transfer molecules to the corpus distal epididymal sperm. A:** Western blot detection of CD9 in Triton X-100 protein extracts of CD9-positive microvesicles isolated from human blood serum. **B:** Relative DilC12 fluorescence intensity on live corpus distal epididymal sperm after coincubation with DilC12-labeled human blood serum or bovine epididymal CD9-positive microvesicles (CD9-MV) in equivalent amount of proteins for 60 min. The maximum fluorescence was considered to be 100%. Results are presented as average ± s.e.m. from three different experiments, *differs significantly *p*<0.05. **C:** Localization of DilC12-labeled molecules from human blood serum exosomes on corpus distal epididymal sperm after coincubation for 60 min at 37°C in sperm medium at pH 6.5. Results are representative of three independent experiments. The experiments were performed with a pool of human blood sera collected from different donors.
(TIFF)

Table S1 **Primers, conditions and products length of the amplified genes.**
(DOC)
