# Supplementary figures and images for: CD9-Positive Microvesicles Mediate the Transfer of Molecules to Bovine Spermatozoa during Epididymal Maturation
Source: PLoS One. 2013 Jun 13;8(6):e65364. doi: 10.1371/journal.pone.0065364 (PMC3681974; doi:10.1371/journal.pone.0065364)

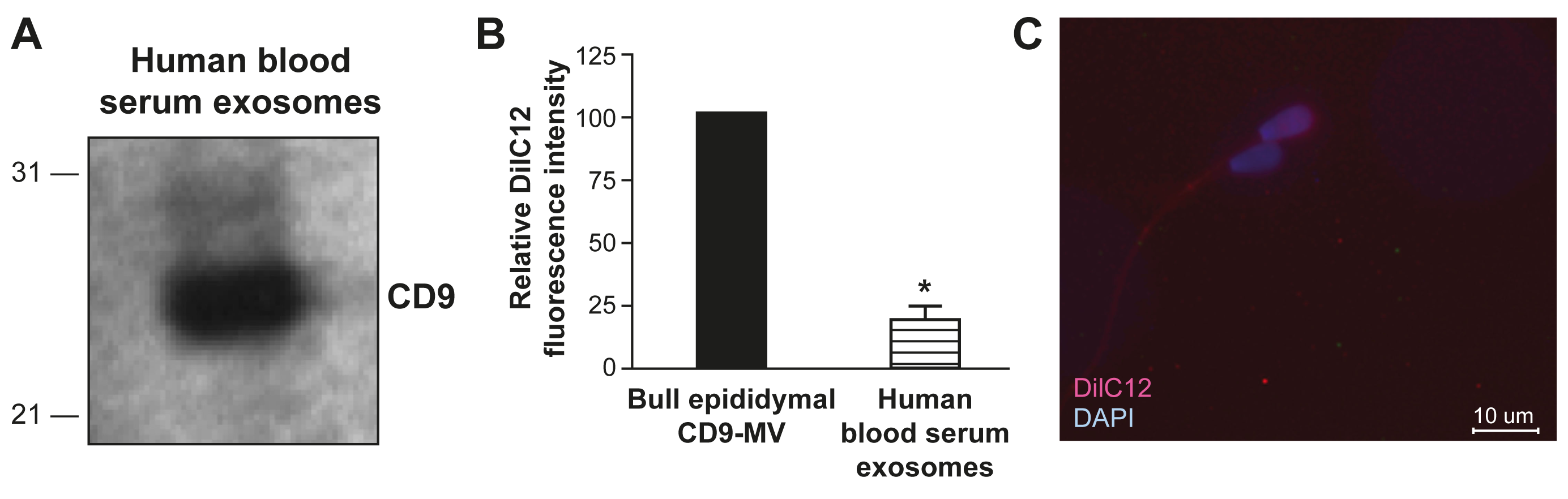

Supplement: Figure S1 — Human blood serum exosomes fail to transfer molecules to the corpus distal epididymal sperm. A: Western blot detection of CD9 in Triton X-100 protein extracts of CD9-positive microvesicles isolated from human blood serum. B: Relative DilC12 fluorescence intensity on live corpus distal epididymal sperm after coincubation with DilC12-labeled human blood serum or bovine epididymal CD9-positive microvesicles (CD9-MV) in equivalent amount of proteins for 60 min. The maximum fluorescence was considered to be 100%. Results are presented as average ± s.e.m. from three different experiments, *differs significantly p<0.05. C: Localization of DilC12-labeled molecules from human blood serum exosomes on corpus distal epididymal sperm after coincubation for 60 min at 37°C in sperm medium at pH 6.5. Results are representative of three independent experiments. The experiments were performed with a pool of human blood sera collected from different donors. (TIFF) [file pone.0065364.s001.tiff]
