# Supplementary material for: CD9-Positive Microvesicles Mediate the Transfer of Molecules to Bovine Spermatozoa during Epididymal Maturation
Source: PLoS One. 2013 Jun 13;8(6):e65364. doi: 10.1371/journal.pone.0065364 (PMC3681974; doi:10.1371/journal.pone.0065364)
Supplement: Table S1 — Primers, conditions and products length of the amplified genes. (DOC) [file pone.0065364.s002.doc]

| Accession number | Gene | Primers | Annealing temperature | Product length |
| --- | --- | --- | --- | --- |
| NM_001114856 | Tubulin | F:5’-TCCATCCACGTTGGCCAGGCT-3’  R:5’-AGCCCCTGTCTCACTGAAGAAG-3’ | 57ºC | 159 bp |
| [NM_001033610.1](http://www.ncbi.nlm.nih.gov/entrez/viewer.fcgi?db=nucleotide&id=75812915) | Cytokeratin 8 | F:5’- TGGCAACATGCAGGGGCTGG -3’  R:5’-CCCTTCCAGGCGGGACTCCA -3’ | 64ºC | 154 bp |
| NM_173900.2 | CD9 | F:5’-AGATCTTCCGAAGCAAATTC -3’  R:5’-CAAAGTTAGTGGCAAAGGAA -3’ | 55ºC | 238 bp |
| [NM_174039.2](http://www.ncbi.nlm.nih.gov/entrez/viewer.fcgi?db=nucleotide&id=31342970) | CD26 | F:5’-GGAATAGCCGTGGCGCCTGT -3’  R:5’-TGTGCTGTGCTGCTGGCGAT -3’ | 61ºC | 296 bp |
| [NM_001206214.1](http://www.ncbi.nlm.nih.gov/entrez/viewer.fcgi?db=nucleotide&id=329664305) | CD224 | F:5’-TGGGCCCGGATCTCCGACAG -3’  R:5’-CGGCTGTTTGCCTGGAGCGA -3’ | 61ºC | 285 bp |
